# Effect of water content on stratum corneum penetration mechanism of W/O type microemulsions†

**DOI:** 10.1039/d3ra02546b

**Published:** 2023-06-12

**Authors:** Erika Nakamura, Hiroki Iwase, Hiroshi Arima-Osonoi, Mina Sakuragi

**Affiliations:** a Faculty of Engineering, Department of Nanoscience, Sojo University 4-22-1 Ikeda, Nishi-ku Kumamoto City 860-0082 Japan d08b0101@nano.sojo-u.ac.jp; b Neutron Science and Technology Center, Comprehensive Research Organization for Science and Society Tokai Ibaraki 319-1106 Japan

## Abstract

The stratum corneum (SC) consists of a lipid layer that forms two types of lamellar structures: short lamellar (S-La) and long lamellar (L-La). It has been reported that S-La contains water phases in the hydrophilic region of the lipids, and that it may play an important role in regulating the water content of the SC. The amount of water in the SC can affect how a drug carrier permeates through the intercellular lipid pathway. To better understand the impact of SC water content on the skin penetration mechanism of a microemulsion (ME), we conducted a study using small-angle X-ray scattering (SAXS), wide-angle X-ray scattering (WAXS), and small-angle neutron scattering (SANS). Our results showed that MEs can enhance skin permeation under humid conditions because the lipid packing structures of the hydrated SC are more disrupted than those of the dry SC. The results also showed that the inner water of MEs was released to the SC when applying MEs to the dry SC, resulting in an increase in the repeat distance of S-La. Conversely, when MEs are applied to hydrated SC, the MEs absorb the water from the SC into their inner phases, causing a decrease in the repeat distance of S-La over time.

## Abstract 2

The stratum corneum (SC) consists of a lipid layer that forms two types of lamellar structures: short lamellar (S-La) and long lamellar (L-La).

## Graphical abstract

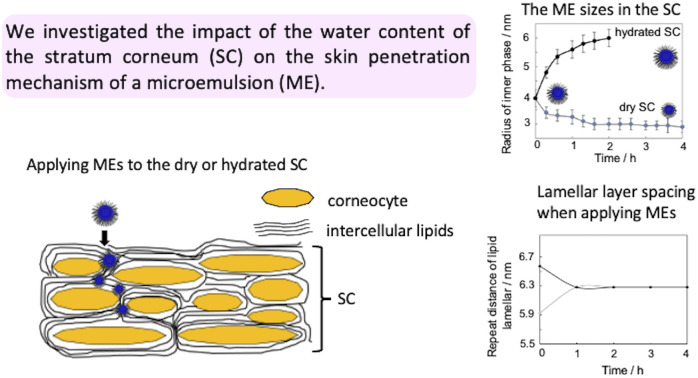

## Introduction

**Fig. 1:**
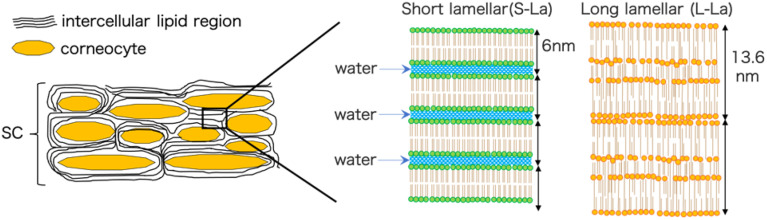
Schematic drawing of the SC and lipid lamellar structures.

The outermost layer of the skin, known as the stratum corneum (SC), is a crucial component in maintaining the skin's barrier function.^1^ The properties of the SC can significantly affect the effectiveness of transdermal drug delivery systems. Typically, small hydrophobic molecules with low molecular weight can penetrate the intracellular lipid layer pathway in the SC, which results in high transdermal permeability.^2,3^ The SC is composed of corneocytes and intercellular lipid layers, which contain long chain ceramides, free fatty acids, and cholesterol.^4^ The lipid layer forms two main types of lamellar structures, short lamellar (S-La) and long lamellar (L-La) structures (Fig. 1). Both mouse and human SC have repeat distances of 6 and 13.5 nm for S-La and L-La, respectively.^5,6^ L-La forms a “sandwich model”^7^ with a broad-narrow-broad sequence of three layers. The hydrocarbon chains in L-La are packed in a hexagonal phase (Hex), with a lattice spacing of 0.41 nm. On the other hand, the hydrocarbon chains in S-La are packed in an orthorhombic phase (OR) with lattice spacings of 0.37 and 0.41 nm.^8^

The water retention function of the SC plays an important role in maintaining the integrity of the skin barrier. The water in the SC is important for SC plasticity and SC morphology.^9^ It also influences the activity of various proteases involved in desquamation and lipid synthesis.^10^ Imokawa *et al.* has been studied about the hydration effect using differential scanning calorimetry (DSC),^11^ and found that the bound water content of the SC is about 33%. Ohta *et al.* conducted a study using small-angle X-ray scattering (SAXS)^12^ to investigate changes in the lipid lamellar structures of the SC at various water contents. The results suggested that the lamellar spacing of S-La increased with higher water content. Conversely, the spacing of L-La did not change between water contents of 0–80% w/w, but the regularity of the lamellar structure did vary. It was proposed that S-La plays an essential role in regulating the water stored in the SC, as it showed a change in the repeat distance, while L-La did not. The water content of the SC can affect the skin penetration mechanism of a drug carrier since the carrier crosses the intercellular lipid lamellar in the SC. However, the interaction mechanism between the drug carrier and intercellular lipids remains unknown when the drug carrier is applied to the SC with different water contents.

The skin penetrations depth of fluorescently labeled drug carrier using fluorescence microscopy has been studied. However, this evaluation using a microscopy cannot elucidate the detailed dynamic skin permeation mechanism at the molecular level. Uchino *et al.* evaluated the interaction mechanism between vesicles and the human SC through synchrotron X-ray scattering.^13^ Their results showed that vesicles affected the S-La structure in the SC, while maintaining their structures. Thus, synchrotron X-ray is a powerful tool to elucidate the dynamic structural change because accurate scattering profiles can be obtained with short exposure times at the molecular level.^14^ Small-angle neutron scattering (SANS) with H/D isotope substitution makes it possible to observe selected components, thus a reliable structural information of the SC intercellular lipids and the drug carrier can be obtained by combining X-ray scattering with SANS.

This study aimed to investigate the impact of SC water content on the skin penetration mechanism of a water-in-oil (W/O) type microemulsion (ME) using SAXS, wide-angle X-ray scattering (WAXS), and SANS. MEs are commonly used as a carrier to enhance the skin permeability of certain drugs.^15–17^ This project is the first attempt to clarify the mechanism of ME penetrating the SC depending on the water contents of the SC.

## Experimental

### Material

Tween-80 and Span-20 were purchased from Sigma Aldrich. Isopropyl myristate (IPM) was obtained from Tokyo Chemical Industry Co., Ltd. The full-thickness skin of seven-week-old male hairless mice (Hos:HR-1, thickness: around 0.7 mm) with subcutaneous fat removed was purchased from Japan SLC, Inc. Fluorescein sodium was obtained from Kanto Chemical Co., Inc.

### ME preparation

MEs were prepared by adding water to the oil phase, which contained the two surfactants Tween-80 and Span-20 in IPM. The samples were then sonicated using a probe sonicator. The weight ratio of Tween-80 and Span-20 were adjusted to 2 : 1, 1 : 1, and 1 : 2. The weight ratio of surfactants, water, and IPM was kept at 20 : 3 : 77.

### Skin penetration experiments

The skin was incubated at 10% humidity for 1 h, 50% humidity for 1 h, and 90% humidity for 2 h. The skin penetration test was performed using Franz diffusion cells (PermeGear, Inc., USA) with a diameter of 5.0 mm. A 10 mM phosphate buffer solution, pH = 7.4, was placed in the receptor chamber and was constantly stirred at 600 rpm at 37 °C. The skin, incubated under each humidity value, was placed on the receptor chamber. Inner phase of MEs was replaced with 0.5 mg mL^−1^ fluorescein sodium aqueous solution. Then, 50 μL of ME labeled with fluorescein sodium was applied to the SC surface. After 48 h, the fluorescein sodium concentration in the receptor chamber was determined using a fluorescence spectrometer (SHIMADZU RF-6000). Fluorescence measurements were conducted at room temperature with a 10 mm path-length quartz cell. The fluorescein sodium was excited at *λ*_ex_ = 485 nm, and its emission was recorded at *λ*_em_ = 535 nm.

### X-ray scattering

The skin was immersed in 0.1% trypsin in pH 7.4 10 mM phosphate buffer at 4 °C overnight, and the SC was separated from the skin after incubation for 4 h at 37 °C. The SC was then immersed in 0.1% trypsin inhibitor and washed with water. To prepare dry SC, three sheets were stacked on a PEEK film and dried in a vacuum chamber overnight. For hydrated SC, the weight of the SC was measured after drying, and then the SC was immersed in water for 3 h. Afterward, the SC was dehydrated under airflow until the water content in the SC reached 50 wt%. SAXS measurements were conducted at the beamline 40B2 at Spring-8, a synchrotron radiation facility in Japan. X-ray diffraction profiles were recorded using a Pilatus detector for SAXS and a flat panel for WAXS. The wavelength was 0.1 nm, and the sample-to-detector distances (SDDs) were approximately 2000 mm for SAXS and 80 mm for WAXS. The dry SC or hydrated SC samples on the PEEK film were set into the sample cell. The cell was made by the University of Kitakyushu Faculty of Environmental Engineering Special Research Laboratory (Machine Center). The SC was measured first, and then MEs were applied to the SC. The scattering patterns were recorded after 1 min and every 5 min consecutively for 40 min. The exposure time was 15 s for SAXS and 4 s for WAXS.

### SANS

SANS measurements were conducted to observe the peaks of L-La and S-La using the small- and wide-angle neutron scattering instrument (TAIKAN) installed on the BL15 beamline at the Materials and Life Science Experimental Facility (MLF), J-PARC, Tokai, Japan.^18^ Neutrons were used in the wavelength range of 0.1–0.7 nm, and the SDD of the detector used was 5.65 m. The stacked SC samples were incubated under 50% RH and 90% RH D_2_O vapor in quartz cells for approximately 10 h. The humidity was controlled using a device that used D_2_O. MEs were applied to the SC incubated under each condition, and the scattering patterns were recorded every hour.

To observe the structural transition of the inner phase of MEs in the SC, SANS measurements were performed on the SANS-U spectrometer installed at JRR-3, Japan Atomic Energy Agency, Tokai, Japan.^19^ The SDD was 2 m, and the neutron wavelength was 0.7 nm with a wavelength distribution (Δ*λ*/*λ*) of 10% full width at half-maximum. The exposure time was 20 min, and all measurements were performed at 25 °C. H_2_O in the inner phase of MEs was replaced with D_2_O (d-ME). For the hydrated SC with H_2_O, the stacked SC sheets were immersed in H_2_O for 4 h. Then, the d-ME penetrating the hydrated SC was measured after excess water on the SC was wiped. For the dry SC, the stacked SC sheets dried using a vacuum pump were used.

## Results

**Fig. 2:**
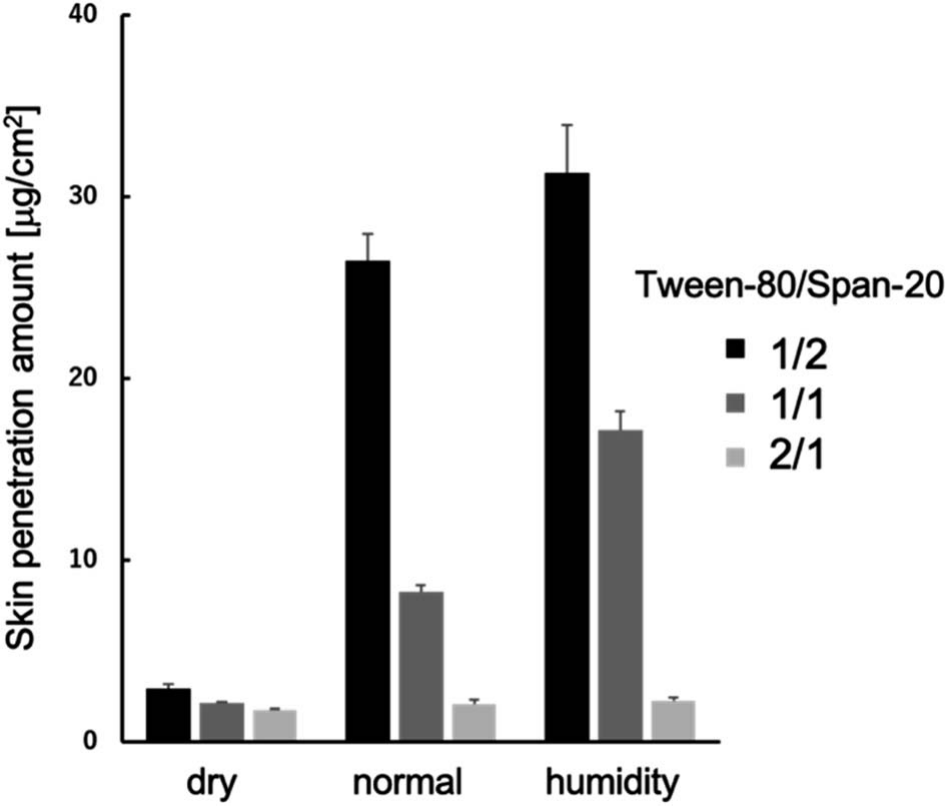
Average penetration of MEs in the hairless mouse skin with different water content (*n* = 3).

**Fig. 3:**
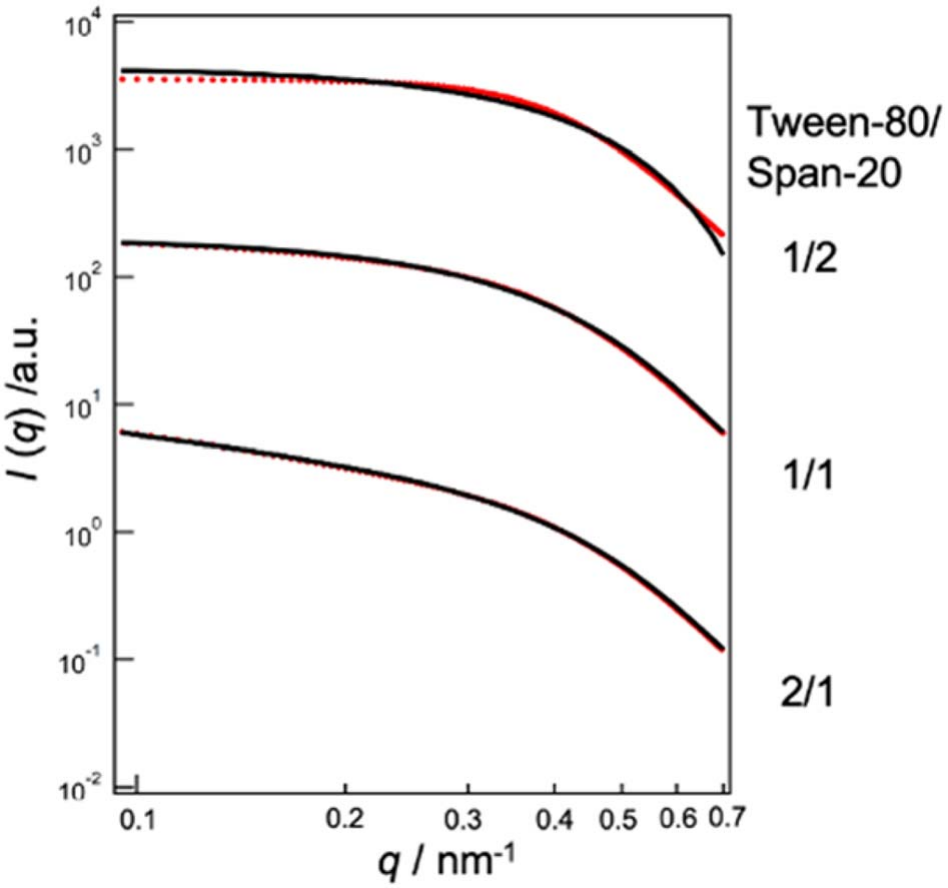
SAXS profiles of MEs at Tween-80/Span-20 = 1/2, 1/1, 2/1. Black lines; theoretical. Red lines; experimental.

We conducted an evaluation of the skin permeation amounts of MEs consisting of Tween-80/Span-20 = 2/1, 1/1, and 1/2 (wt/wt), with varying skin water contents (Fig. 2). Our results indicated that for all MEs, skin permeabilities increased as skin water content increased. The ME with a Tween-80/Span-20 ratio of 1/2 exhibited the highest skin permeability among the prepared MEs. SAXS profiles of these MEs revealed that the MEs with a Tween-80/Span-20 ratio of 2/1 formed cylindrical structures with a cross-sectional diameter of 7.0 nm, while the MEs with ratios of 1/1 and 1/2 formed spherical structures with diameters of 11.4 and 10.8 nm, respectively (Fig. 3).

Consequently, we investigated the skin permeation mechanism of the ME with a Tween-80/Span-20 ratio of 1/2, which exhibited the highest skin permeability, in subsequent steps.

**Fig. 4:**
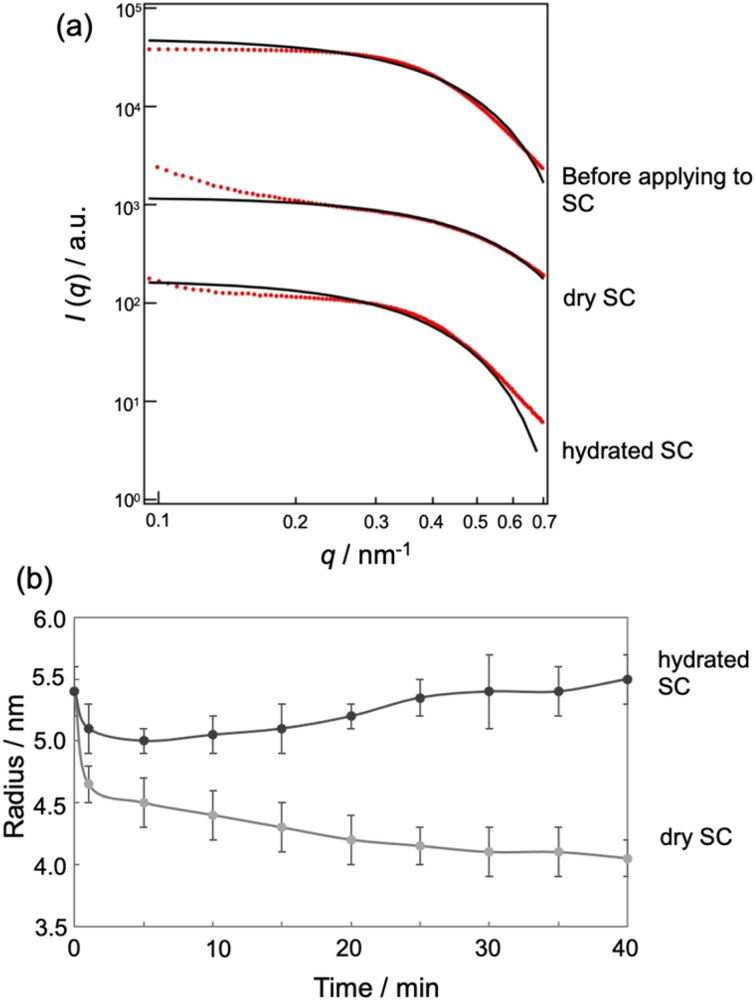
(a) SAXS profile (red) and theoretical curve (black) of MEs in SC with different water contents. (b) Time course of ME sizes in SC with different water contents.

Fig. 4a displays SAXS profiles of the MEs before and after application to the dry or hydrated SC for 40 min. The SAXS profile of the MEs before application to the SC satisfies *I*(*q*) ∼ *q*^*α*^ with *α* = 0 and shows a dramatic decrease in intensity for *q* > 0.4 nm^−1^, indicating the presence of a spherical structure. *q* is the magnitude of the scattering vector defined as *q* = 4π sin(*θ*/2)/*λ* (*θ* and *λ* represent the scattering angle and wavelength, respectively). Upon application to the dry or hydrated SC, the slope in the low *q* region increased due to the small amounts of aggregates present. In the *q* range of 0.2–0.35 nm^−1^, we also observed *I*(*q*) ∼ *q*^*α*^ with *α* = 0 and a dramatic decrease in intensity in the high *q* region, indicating the presence of a spherical structure. To analyze the SAXS profiles, we fitted them using a scattering function of a sphere as given by^20^1
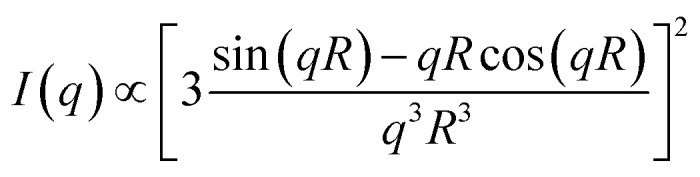
where *R* indicates the radius of a sphere. The agreement between the experimental and fitted data was quite good in the low *q* region, but not in the high *q* region where the slope increased due to the hydrocarbon chains of the surfactants behaving as a Gaussian chain.^21,22^ In this study, only the sizes of the MEs were calculated from low *q* region.

Fig. 4b shows the time-dependent changes in ME size in the SC, as influenced by SC water content. In drying conditions, the ME size decreased with time, while for hydrated conditions, the size increased. The decrease in particle size for the drying SC may be attributed to the release of water from the MEs into the SC. Conversely, the increase in size for the hydrated SC may be due to the absorption of water from the SC into the MEs.

**Fig. 5:**
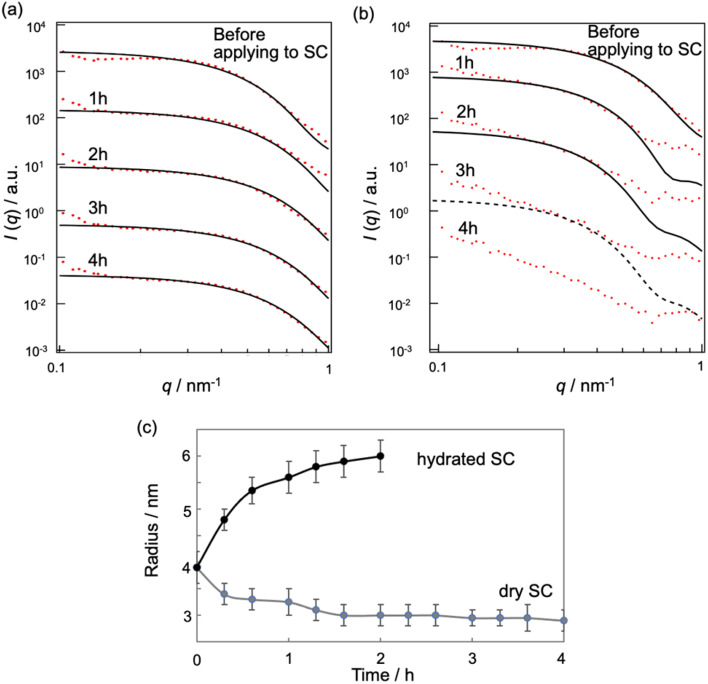
SANS profile (red) and theoretical curve (black) of d-MEs in dry SC (a) and in hydrated SC (b). (c) Time course of the inner size of the d-MEs applied to SC with different water contents.

We can investigate the structural transition of the inner phase of MEs in the SC by using SANS, in which H_2_O in the inner phase of MEs is replaced with D_2_O, denoted by d-MEs. Fig. 5a and b depict the time changes of SANS profiles of d-MEs before and after application to the (a) dry SC and (b) hydrated SC, respectively. In Fig. 5a, a plateau region at low *q* was observed before and after applying d-MEs to the dry SC, indicating that d-MEs maintained their spherical structure for at least 4 h. By fitting the data using eqn (1), we obtained the sizes of the inner phase of d-MEs. The agreement between the experimental and fitted data was excellent in all regions because only the inner phase was replaced with deuterium, and the hydrocarbon chain of surfactants, which affects scattering in the high *q* region, was not observed. This finding suggests that the inner phase forms a shape close to a sphere defined by eqn (1).

In Fig. 5c, the temporal evolution of the radii of the inner phase of d-MEs is presented for both dry and hydrated SC conditions. For the dry SC, the inner phase radii decreased over time, indicating the release of D_2_O molecules from the inner phase into the SC. This result is supported by the absolute intensity decrease observed in Fig. S1a.†

Fig. 5b shows SANS profiles of d-MEs before and after applying them to the hydrated SC. The results indicate that d-MEs maintained their spherical structures for at least 2 h after application to the SC. The experimental curves were not fitted with theoretical curves in the high *q* region because d-MEs absorbed water in the SC in the inner phase, leading to incoherent scattering effects. According to our fitting analysis using eqn (1), the size of the inner phase of d-MEs increased with time (Fig. 5c). We also could not fit the experimental curves after 3 h using eqn (1) because of incoherent scattering effects of H_2_O adsorbed in the inner phase of d-MEs. The decrease of absolute intensity with time in Fig. S1b† is also due to the same reason. These SANS results are consistent with the SAXS results.

**Fig. 6:**
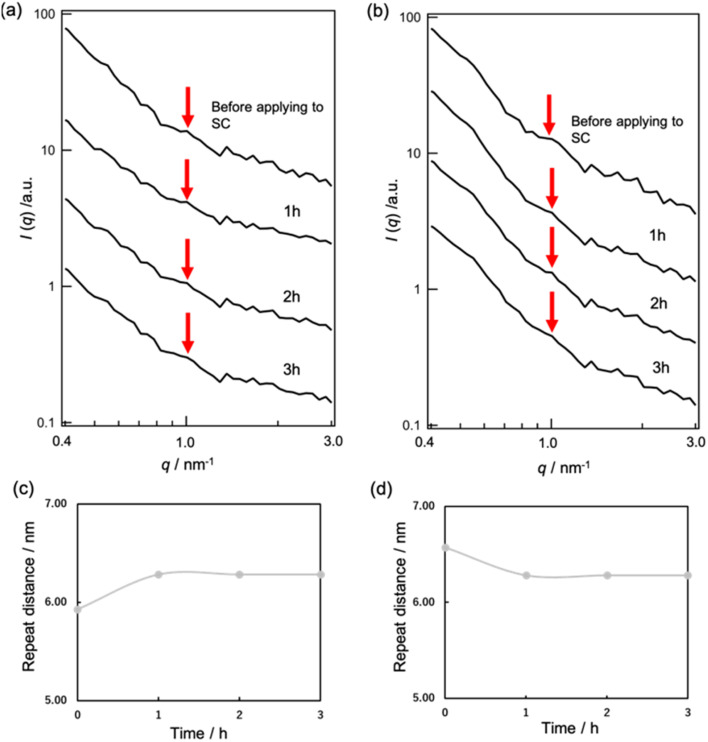
SANS profile of SC under (a) 50% RH D_2_O and (b) 90% RH D_2_O conditions when applying MEs. Changes in the repeat distances of S-La when applying MEs to SC under (c) 50% RH D_2_O and (d) 90% RH D_2_O conditions.

We proceeded to observe the peaks of S-La. However, due to the high intensity of the MEs' form factor, we were unable to observe these peaks through SAXS experiments with MEs present. To address this issue, we conducted SANS experiments under D_2_O vapor conditions. When the SC samples were exposed to 50% and 90% RH D_2_O vapor, peaks appeared at approximately 1.06 nm^−1^ (*d* = 5.93 nm) and 0.956 nm^−1^ (*d* = 6.57 nm), respectively (Fig. 6a and b).

Upon applying MEs to the SC under the 50% RH D_2_O condition, we observed a shift in the peak position to a lower *q* value with time, indicating an increase in the repeat distance of S-La from 5.93 to 6.28 nm (Fig. 6c). These results suggest that MEs released inner water into the SC lipid lamellar phase, causing the repeat distance to increase.

Conversely, when MEs were applied to the SC under the 90% RH D_2_O condition, the peak of S-La shifted to a higher *q* value, and the repeat distance of S-La decreased from 6.57 to 6.28 nm (Fig. 6d). These observations are consistent with the SANS experimental results obtained when applying d-ME to SC, indicating that the decrease in the repeat distance may be due to the water molecules in the SC lamellar being taken into the inner phase of MEs.

**Fig. 7:**
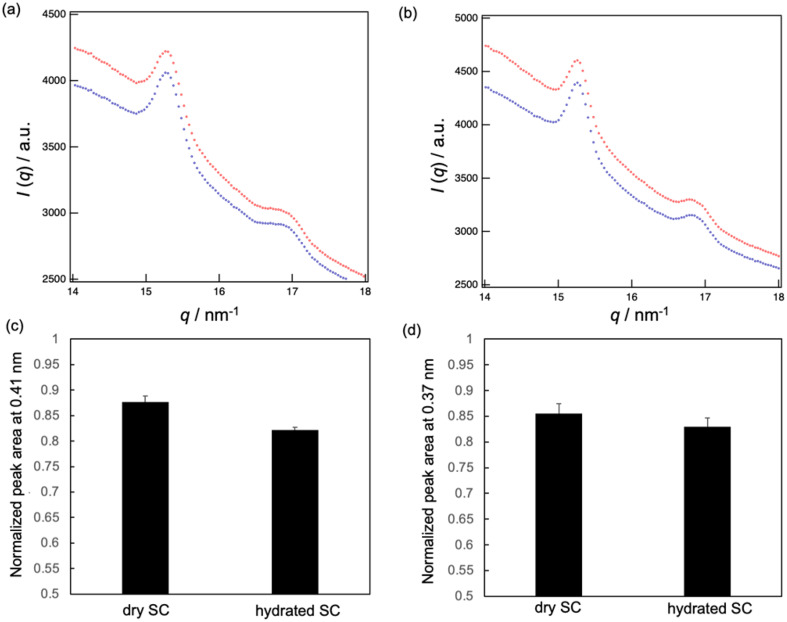
WAXD profiles of the SC at 1 min (blue) and 40 min (red) after applying MEs to the dry SC (a) and hydrated SC (b). Normalized peak area ratios of (c) 0.41 nm and (d) 0.37 nm after 40 min to 1 min after applying MEs (*n* = 6).

Finally, the effect of applying MEs on the lateral lipid packing structure of hydrated and dry SC was analyzed by WAXS. Two peaks were observed in the wide-angle region: one at 0.41 nm and the other at 0.37 nm (Fig. 7a and b). The peak at 0.41 nm corresponds to the lattice spacings of OR and Hex, while the peak at 0.37 nm corresponds to that of OR.^8,15,23^ These peak intensities decreased after applying MEs, while the peak position did not change over time (Fig. 7a and b). We calculated the average peak area ratios at 40 min to 1 min after applying MEs (Fig. 7c and d) and found that the peak area ratios for the hydrated SC were lower than those for the dry SC. This indicates that the lipid packing structures in the hydrated SC were more disturbed by the MEs than those in the dry SC. Therefore, MEs can permeate the hydrated SC more effectively than the dry SC.

## Discussion

MEs consisting of Tween-80/Span-20 = 2/1, 1/1, and 1/2 (wt/wt) were prepared. We found that their particle sizes increased with increase in the Tween-80/Span-20 (Fig. 3). The larger chemical structure of Tween-80 compared to Span-20 likely led to the composition containing more Span-20 to form smaller ME particles. This tendency is the same as in previous studies.^22,24^ These smaller MEs appeared to permeate the narrow intercellular space in the SC more easily than larger MEs (Fig. 2), which is consistent with our previous findings.^25^ However, the smaller size does not necessarily mean better penetration. The composition and shape of the ME as well as the location of the drug within the ME also effect skin penetration.^26^ Our results also showed that skin permeabilities increased as skin water content increased (Fig. 2). It has been reported that an ME with high water content in its inner phase can have a hydration effect, enhancing skin penetration.^27^ PEGylation of surfactant-containing liposomes can increase the skin permeation of some drugs because PEG-bonded water increases the SC hydration.^28^ Thus, hydrated SC is known to enhance the skin permeation of the substances. The reason is that SC lipid packing structures are disrupted by the hydration when MEs penetrate the SC, as shown in Fig. 7, making it easier for drug carriers to permeate the intercellular pathway of the SC.

The change in the sizes of MEs at Tween-80/Span-20 = 1/2 when applied to the SC was evaluated by SAXS and SANS. For fitting analysis of the SAXS profile using a spherical equation, the agreement between the experimental and theoretical was not good in the high *q* region because the power law slope obeys *q*^−2^ at a high *q* value (Fig. 4a). Eqn (1) is assumed have a sharp interface between MEs and the solvent and the power law slope in the high *q* region should obey *q*^−4^. In our system, hydrocarbon chains in the outer phase of MEs seem to behave as Gaussian chains.^22,29,30^ Alternatively, when MEs form other shapes such as an ellipsoid, the power law slope in the wide-angle region does not obey *q*^−4^.^31^ However, since only the D_2_O phase of MEs could be observed for SANS, the agreement between the experimental and fitted data was quite good (Fig. 5a). This suggests that MEs form a spherical structure.

Our SAXS analysis show that the ME size decreased slightly soon after its application to the hydrated SC, and then it increased with time after 5 min (Fig. 4c). Immediately after application to the SC, the structure of MEs may be slightly disturbed due to their interactions with SC lipids. After that, the ME increases in size while taking in water in the SC. Although we could not observe the ME size soon after application to the hydrated SC due to the exposure time for SANS, the results after 20 min were consistent with our SAXS results (Fig. 5c). For dry SC, the decrease in ME size was observed in SAXS and SANS (Fig. 4c and 5c). This is due to the release of water from the MEs into the SC. The fact that incoherent scattering effects increased over time also indicates that H_2_O molecules of the SC were taken into the MEs (Fig. 5b and S1†).

The S-La peak in the SC was evaluated using SANS under 50% and 90% RH D_2_O vapor. The peaks appeared at approximately 1.06 nm^−1^ (*d* = 5.93 nm) for 50% RH and 0.956 nm^−1^ (*d* = 6.57 nm) for 90% RH (Fig. 6a and b). These peaks correspond to the repeat distance of a portion occupied by D_2_O in the hydrophilic phase of S-La. The observed increase in repeat distance at the hydrated SC condition compared to the dry SC condition is in agreement with the swelling behavior of the water layer in the S-La reported by Ohta *et al.*^12^ and Bouwstra *et al.*^32^

When MEs were applied to the SC in 50% RH, an increase in the repeat distance of S-La from 5.93 to 6.28 nm was observed (Fig. 6c) because MEs released inner water into the SC lipid lamellar phase. At 90% RH, the repeat distance of S-La decreased from 6.57 to 6.28 nm (Fig. 6d) due to the water molecules in the SC lamellar being taken into the inner phase of the MEs. Uchino *et al.* investigated the interaction between vesicles and the human SC by synchrotron X-ray scattering^13^ and monitored the changes in the small-angle X-ray diffraction peaks of the human SC after buffer control and vesicle application over time. They concluded that vesicles affected the S-La structure in the SC, maintaining their structures. They also suggested that the change in the S-La structure occurred through water penetration. Hathout *et al.* evaluated the uptake of MEs at various components into the SC by infrared spectroscopy.^33^ Their results showed that SC hydration increased proportionally to the water content of the MEs, and the SC structure was disturbed by all components of MEs. Our results showing that the MEs affect structural change in S-La are consistent with their results.

## Conclusions

In this study, we investigated how water content affects the penetration mechanism of W/O type MEs in the SC. Using hairless mouse skin, we observed that skin permeability increased with increasing skin water content and smaller MEs had higher permeability compared to larger MEs. We then selected the smallest spherical MEs for further experiments.

We then examined the structural changes of MEs in the SC and the variation in the repeat distance of S-La upon application of MEs, depending on the SC water content. SAXS analysis revealed that the radii of spherical MEs decreased in the drying SC while they increased in the hydrated SC. Using SANS, we successfully observed the scattering from the internal phases of MEs penetrating the SC. Our results suggested that for the drying SC, MEs might release inner water into the SC, whereas for the hydrated SC, water in the SC might be taken up into the MEs. These findings were consistent with the SAXS results. Furthermore, we replaced the H_2_O molecules in the hydrophilic phase of S-La with D_2_O molecules by the supply of D_2_O vapor, and the D_2_O phase in the SC when ME was applied. The results indicated that the water in S-La was taken up by the MEs for the hydrated SC, resulting in a decrease in the repeat distance, whereas the repeat distance increased for the drying SC, possibly because MEs released inner water into the S-La region.

Finally, we analyzed the peak areas derived from SC lipid packing structures when applying MEs and found that the lipid packing structures of the hydrated SC were more disrupted by penetrating MEs compared to the dry SC. We are currently evaluating the skin penetration mechanism of MEs with different structures.

This research establishes an essential technique for observing the SC permeation mechanism of ME. This allows us to propose a skin permeation carrier formulation suitable for skin conditions with different water contents.

## Author contributions

EN: data curation, structural analysis, investigation, methodology, writing – original draft. HI: methodology, writing – review and editing. HAO: methodology, writing – review and editing. MS: investigation, methodology, funding acquisition, project administration, resources, supervision, writing – original draft, review, and editing.

## Conflicts of interest

The authors declare no conflicts of interest.

## Supplementary Material

RA-013-D3RA02546B-s001
