# Supplementary material for: Effect of water content on stratum corneum penetration mechanism of W/O type microemulsions
Source: RSC Adv. 2023 Jun 12;13(26):17742–9. doi: 10.1039/d3ra02546b (PMC10259503; doi:10.1039/d3ra02546b)
Supplement: RA-013-D3RA02546B-s001 [file RA-013-D3RA02546B-s001.pdf]

Electronic Supplementary Material (ESI) for RSC Advances.
This journal is © The Royal Society of Chemistry 2023

Supporting Information

Effect of water content on stratum corneum penetration mechanism of W/O type microemulsions

Erika Nakamura,[a] and Hiroki Iwase,[b] and Hiroshi Arima-Osonoi,[b] and Mina Sakuragi*[a]

[a]Faculty of Engineering, Department of Nanoscience, Sojo University, 4-22-1 Ikeda, Nishi-ku, Kumamoto City 860-0082

[b]Neutron Science and Technology Center, Comprehensive Research Organization for Science and Society, Tokai, Ibaraki, 319-1106, Japan

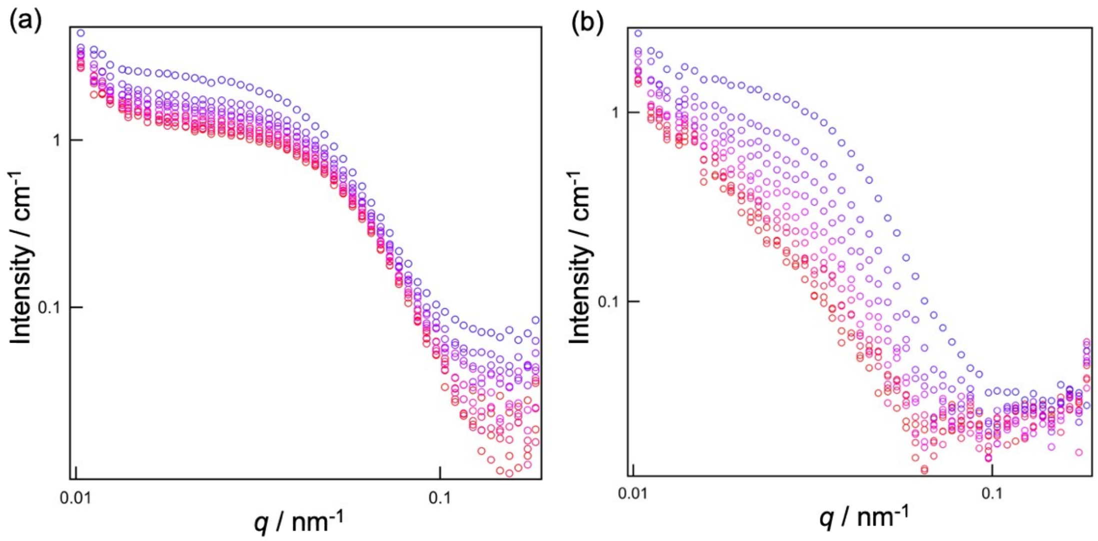

Fig. S1 Change of SAXS profiles from blue to red over time in d-MEs applied to (a) the dry SC and (b) hydrated SC.
